# *Micrococcus luteus*-derived extracellular vesicles attenuate neutrophilic asthma by regulating miRNAs in airway epithelial cells

**DOI:** 10.1038/s12276-022-00910-0

**Published:** 2023-01-13

**Authors:** Soyoon Sim, Dong-Hyun Lee, Kwang-sun Kim, Hyeon Ju Park, Yoon-Keun Kim, Youngwoo Choi, Hae-Sim Park

**Affiliations:** 1 Department of Allergy and Clinical Immunology, Ajou University School of Medicine, Suwon, Korea; 2 Department of Biomedical Sciences, Graduate School of Ajou University, Suwon, Korea; 3 Department of Chemistry and Chemistry Institute for Functional Materials, Pusan National University, Busan, Korea; 4 MD Healthcare Inc., Seoul, Korea

**Keywords:** Chronic inflammation, Acute inflammation

## Abstract

Bacterial extracellular vesicles (EVs) have been shown to regulate various pulmonary diseases, but their functions in asthma remain uncertain. To demonstrate the clinical significance of *Micrococcus luteus*-derived EVs (MlEVs) in asthma, we enrolled 45 asthmatic patients (20 patients with neutrophilic asthma [NA], 25 patients with eosinophilic asthma [EA]) and 40 healthy controls (HCs). When the prevalence of IgG1 and IgG4 specific to MlEVs was evaluated in serum by ELISA, lower levels of MlEV-specific IgG4 (but not IgG1) were noted in asthmatic patients than in HCs. Among asthmatic patients, significantly lower levels of MIEV-specific IgG4 were noted in patients with NA than in those with EA. Moreover, there was a positive correlation between serum MlEV-specific IgG4 levels and FEV_1_ (%) values. In asthmatic C57BL/6 mice, MlEVs significantly attenuated neutrophilic airway inflammation by reducing the production of IL-1β and IL-17 in bronchoalveolar lavage fluid as well as the number of group 3 innate lymphoid cells (ILC3s) in lung tissues. To clarify the functional mechanism of MlEVs in NA, the effect of MlEVs on airway epithelial cells (AECs) and immune cells was investigated ex vivo. According to microarray analysis, MlEVs upregulated hsa-miR-4517 expression in AECs. Moreover, this miRNA could suppress IL-1β production by monocytes, resulting in the inhibition of ILC3 activation and neutrophil recruitment. These findings suggest that MlEVs could be a novel therapeutic agent for managing unresolved NA by regulating miRNA expression in AECs.

## Treatment of asthma using extracellular vesicles from bacteria

Tiny membrane-bound sacs released by bacteria and containing a variety of biomolecules show potential as a novel treatment for asthma. These sacs, known as extracellular vesicles (EVs), are released by all living cells. Some bacterial EVs have been found to influence the immune response in animal models and asthmatic patients. Researchers in South Korea led by Youngwoo Choi and Hae-Sim Park at Ajou University School of Medicine, Suwon, demonstrated the beneficial effects of EVs from *Micrococcus luteus* bacteria on neutrophilic inflammation in patients with neutrophilic asthma. Studies in mice using cultured airway and immune systems cells revealed details of the mechanism of the EV’s effects. Bacterial EV biomolecules increased the production of regulatory RNA molecules, which inhibited cellular signaling pathways involved in neutrophilic asthma.

## Introduction

Asthma is a multifactorial disorder characterized by chronic airway inflammation with complex interactions between genetic and environmental factors^1^. For decades, allergen exposure has been widely accepted to induce adaptive immunity in asthma pathogenesis^2^. In particular, eosinophils have been recognized as the main effector cells contributing to persistent airway inflammation^3^. Nevertheless, current therapeutic agents targeting the Th2-driven immune response have limitations, as 5–10% of asthmatic patients persist with an uncontrolled status^4,5^. Neutrophilic asthma (NA) is a severe asthma phenotype related to the innate immune response induced by viruses or bacteria^6^. Although neutrophils play a pivotal role in host defense^7^, their massive infiltration in the airways induces frequent asthma exacerbation and steroid resistance.

Accumulating evidence has shown that distinct bacterial burdens contribute to the pathogenesis of NA, suggesting the importance of the microbiome in the innate immune system of the airways^8,9^. In particular, airway epithelial cells (AECs) are critical for initiating neutrophil recruitment to the airways by releasing IL-8 in response to invading microbes^10^. Moreover, various innate immune cells, such as dendritic cells, monocytes, and innate lymphoid cells (ILCs), could affect the homeostasis of the airway microbiome^11^. Additionally, the pathological role of ILC3s is highlighted because they are a major source of IL-17 and IL-22, which enhance airway inflammation and remodeling in asthma^12^. Furthermore, the secretion of these cytokines could be amplified by their IL-1β-dependent interactions with monocytes or macrophages^13^. Therefore, manipulation or regulation of microbial communities may be an applicable approach for modulating innate immune responses in NA.

Extracellular vesicles (EVs) are membranous particles harboring various molecules, including lipids, proteins, and nucleic acids. They are released from all living cells under physiological and pathological states^14^. In particular, the role of bacterial EVs in modulating host immunity in various diseases has been highlighted^15–18^. Recently, metagenomic data analyses have revealed the significance of bacterial EVs found in serum or urine as novel biomarkers^19–21^. Moreover, a low prevalence of *Micrococcus luteus*-derived EVs (MlEVs) was observed in serum from asthmatic patients^22^. *M. luteus* is a commensal species in human skin and the upper respiratory tract^23^. Although this bacterium has been considered a nonpathogen, it is occasionally reported to be involved in infectious or inflammatory diseases^24–26^.

Here, we aimed to demonstrate the significance of MlEVs in NA by (1) evaluating the relative abundance of EV-specific antibodies in the serum of asthmatic patients with different inflammatory phenotypes and (2) investigating the effect of MlEVs on airway immune responses in vivo and ex vivo.

## Materials and methods

### Bacterial EV isolation and characterization

*M. luteus* (Korean Culture Center of Microorganisms, Seoul, Korea) in Ediable LP medium (MD Healthcare Inc., Seoul, Korea) and *Escherichia coli* (American Type Culture Collection, Manassas, VA, USA) in LB medium (ThermoFisher Scientific, Waltham, CA, USA) were each cultured under aerobic conditions until the optical density reached 1.5 at 600 nm. For EV isolation, each culture medium was centrifuged at 10,000 ×*g* for 20 min, and the supernatant was filtered through a 0.45-μm vacuum filter. The filtrate was enriched using a QuixStand (GE Healthcare, Little Chalfont, UK) and subsequently filtered through a 0.22-μm bottle-top filter (Sigma-Aldrich, St. Louis, MO, USA). Then, the filtrate was pelleted by ultracentrifugation in a 45–Ti rotor (Beckman Coulter, Fullerton, CA, USA) at 150,000 ×*g* for 2 hours at 4 °C. The final pellets were resuspended in phosphate-buffered saline (PBS) and stored at −80 °C. EV shape was observed using a JEM1011 microscope (JEOL, Akishima, Japan). EV size was measured using a Zetasizer Nano S (Malvern Instruments, Malvern, UK). For proteomic analysis, tryptic peptides were loaded onto a trap column packed with a resin and eluted using a linear gradient from 5–40% solvent B (0.1% formic acid in acetonitrile). The eluted peptides were separated on an analytical column packed with a resin and sprayed into a nano ESI source using an electrospray. A Q Exactive mass spectrometer was operated to choose the most abundant precursor ions using a survey scan.

### Patient recruitment and clinical parameters

This study was approved by the Institutional Review Board of Ajou University Hospital (AJIRB-GEN-SMP-13-108; AJIRB‐BMR‐SUR‐15‐498). All the study subjects provided written informed consent at the time of recruitment. Here, 45 asthmatic patients and 40 healthy control subjects (HCs) diagnosed by allergy specialists based on clinical histories such as recurrent cough, shortness of breath, recurrent wheezing, chest tightness, and evidence of airway obstruction were recruited. Severe asthma was defined according to the GINA guidelines^27^. Moreover, asthmatic patients were classified into EA and NA groups according to the percentage of sputum eosinophils (3%) and neutrophils (65%). Atopy was defined as at least one positive result in skin prick tests using inhaled allergens (Bencard, Bradford, UK). Serum total IgE was measured using the ImmunoCAP system (ThermoFisher Scientific). The levels of eosinophil cationic protein (ECP) and myeloperoxidase (MPO) in serum were measured using respective ELISA kits (R&D Systems, Minneapolis, MN, USA) according to the manufacturer’s recommendations.

### Evaluation of the abundance of EV-specific antibodies in serum

To evaluate the levels of EV-specific IgG subclasses in serum from the study subjects, MlEVs or *Escherichia coli*-derived EVs (EcEVs) were coated at 100 ng/mL on a 96-well plate (ThermoFisher Scientific) for 12 h at 4 °C. The plate was blocked with 1% bovine serum albumin (Sigma-Aldrich) and incubated with serum from the study subjects. Then, a peroxidase-conjugated anti-human IgG1 or IgG4 antibody (Sigma-Aldrich) was added. The reaction was induced by 3,3′,5,5′-tetramethylbenzidine (BD Biosciences, San Diego, CA, USA) and stopped by using a stop solution. The intensity was evaluated by using a microplate reader (BioTek, Santa Clara, CA, USA) at a wavelength of 450 nm.

### Mouse experiments and evaluation

All the animal studies were approved by the Institutional Animal Care and Use Committee of Ajou University (IACUC-2016-0022). To induce NA, male 6-week-old C57BL/6 mice (Orient BIO, Seongnam, Korea) were intranasally treated with 75 µg of ovalbumin (OVA; Sigma-Aldrich) and 10 µg of lipopolysaccharide (LPS; Sigma-Aldrich) for sensitization on Days 0, 1, 2, and 7. Then, sensitized mice were intranasally treated with 50 µg of OVA in 20 µL of PBS for challenges on Days 14, 15, 16, 17, and 18. During challenges, mice were intranasally treated with 0.2 mg/mouse dexamethasone (Dex; Sigma-Aldrich) or 1 μg/mouse MlEVs at the same time as OVA administration. To measure immune cell numbers in bronchoalveolar lavage fluid (BALF), Diff-Quick staining (Dade Behring, Dudingen, Switzerland) was conducted. Levels of IL-1β and IL-17 were quantified by using ELISA kits (R&D Systems). For histological analysis, the lung tissues were stained with H&E. In the lungs, the expression levels of NLRP3 (Cell Signaling Technology, Danvers, MA, USA), T-bet (Abcam, Cambridge, UK), and ROR-γt (Invitrogen, Waltham, MA, USA) were measured. For flow cytometric analysis of ILC3s, perfused lungs were chopped into small pieces. After incubation with collagenase type IV (ThermoFisher Scientific) for 30 min, the tissues were sequentially filtered through 100-μm and 40-μm nylon cell strainers (SPL Life Science, Pocheon, Korea). Then, red blood cells were eliminated using a lysis solution (Miltenyi Biotec, Auburn, CA, USA). After being washed with PBS, the cells were incubated with antibodies as follows: anti-lineage (BD Biosciences), anti-CD45, anti-CD278 (BioLegend, San Diego, CA, USA), anti-CD117, and anti-CD127 (ThermoFisher Scientific). The analysis was performed by a FACSAria III (BD Biosciences), and graphs were produced using FlowJo software (Tree Star, Ashland, OR, USA).

### In vivo fluorescence imaging

To detect MlEVs in organs, MlEVs were incubated with 5 µM Cy7 mono NHS ester (GE Healthcare) for 1 h at 37 °C, and Cy7-labeled MlEVs were isolated by ultracentrifugation. Then, 10 μg of Cy7 mono NHS ester-labeled MlEVs were administered intranasally to the mice. After 6 h, the mice were sacrificed, and Cy7 fluorescence in extracted organs was detected using a Davinch-Invivo system (Davinch-Invivo Fluoro Chemi, Seoul, Korea).

### Human AEC stimulation

A549 cells (American Type Culture Collection) were purchased and cultured in RPMI-1640 medium (ThermoFisher Scientific) with 10% fetal bovine serum (ThermoFisher Scientific). Then, the A549 cells were treated with 1 μM Dex or 1 μg/mL MlEVs in the presence of 10 μg/mL LPS for 24 h. IL-8 levels in culture supernatants were measured using an ELISA kit (R&D Systems), and protein expression in the cells was evaluated using the following antibodies: phospho-p65 (Cell Signaling Technology), p65 (Abcam), and actin (Santa Cruz, Dallas, TX, USA). AEC-derived EVs (AEC-EVs) were isolated by ultracentrifugation and confirmed by EV markers, including ALG-2-interacting protein X (ALIX; Santa Cruz) and tumor susceptibility 101 (TSG101; Santa Cruz). Moreover, EV protein patterns were analyzed by sodium dodecyl sulfate-polyacrylamide gel electrophoresis.

### Microarray analysis for library production and miRNA identification

To generate a library, adapter ligation, reverse transcription, PCR amplification, and pooled gel purification were conducted. The RNA 3′ adapter was specifically modified to target microRNAs and other small RNAs that have a 3′ hydroxyl group resulting from enzymatic cleavage by Dicer or other RNA-processing enzymes. The adapters were ligated to either end of the RNA molecule, and an RT reaction was used to create single-stranded cDNA. The cDNA was then PCR amplified using a common primer and a primer containing one of 48 index sequences. The introduction of the index sequence at the PCR step separated the indices from the RNA ligation reaction. This design allows for the indices to be read using a second read and significantly reduces bias compared to that approaches that include the index within the first read.

The library was gel purified by BluePippin and validated by checking the size, purity, and concentration on an Agilent Bioanalyzer. The library was quantified using KAPA Library Quantification Kits for Illumina Sequencing Platforms according to the qPCR Quantification Protocol Guide (Kapa Biosystems, Wilmington, MA, USA). The indexed library was then submitted to an Illumina HiSeq2500 (Illumina, San Diego, CA, USA) instrument to generate 51-base reads. Adapters in the raw reads were trimmed using the cutadapt program^28^. If a sequence was matched to more than the first 5 bp of the 3′ adapter for read 1 or the 5′ adapter for read 2, it was regarded as an adapter sequence and then trimmed from the reads. Trimmed reads whose lengths were longer than 18 bp were selected for the determination of mapping reliability. Then, the remaining reads were classified as nonadapter reads, whose adapter sequences were not sequenced. To minimize the sequence redundancy for computational efficiency, trimmed reads were clustered by sequence. A unique cluster consisted of the reads whose sequences and lengths were the same. To eliminate rRNA, we excluded reads that were aligned to the 45S pre-rRNA and mitochondrial rRNA of humans. To identify known miRNA reads, the sequence alignment and detection of known and novel microRNAs were performed using the miRDeep2 software algorithm^29^. Therefore, rRNA-filtered reads were aligned to the mature and precursor miRNAs of humans obtained from miRBase v22.1 using the miRDeep2 quantifier module^30^. To predict novel miRNAs, the reference genome of humans, release hg19, was retrieved from RefSeq. The reference genome was indexed, and rRNA-filtered reads were mapped to it using Bowtie (1.1.2)^31^. Novel microRNAs were predicted from the mature, star, and loop sequences according to the RNAfold algorithm using miRDeep2. Uniquely clustered reads were then sequentially aligned to the reference genome, miRBase v22.1, and noncoding RNA database RNAcentral release 14.0 to identify known miRNAs and other types of RNA for classification^32^.

### Human peripheral monocyte isolation and miRNA transfection

Blood from asthmatic patients was collected into vacutainer tubes containing acid citrate dextrose solution (BD Biosciences) and layered in Lymphoprep solution (Axis-Shield, Oslo, Norway) followed by centrifugation at 879×*g* at 20 °C for 25 min. Then, monocytes in the cloudy layer of peripheral blood mononuclear cells were collected using a monocyte isolation kit (Miltenyi Biotec). Monocytes were added (1 × 10^6^) to a 24-well plate in RPMI 1640 medium supplemented with 2% fetal bovine serum and treated with 1 µM Dex or 1 µg/mL AEC-EVs for 48 hours in the presence of 10 µg/mL LPS. For transfection with miRNA mimics, monocytes (1 × 10^6^) were seeded on a 24-well plate. Transfection was conducted using the following synthetic miRNA mimics: miRNA-197-5p, 579-3p, 3065-5p, and 4517 (MyBioSource, San Diego, CA, USA) as well as 1272 and 1291 (Sigma-Aldrich). Then, 50 nM synthetic miRNA mimics were transfected into cells by using Lipofectamine™ 3000 Transfection Reagent (ThermoFisher Scientific). In monocytes, the expression of NOD-, LRR-, and pyrin domain-containing protein 3 (NLRP3) was observed by western blot analysis. The levels of IL-1β in the supernatants were measured using an ELISA kit (R&D Systems).

### Quantitative analysis of miRNA expression in plasma

For the analysis of miRNA expression in plasma, total RNA was extracted using a miRNeasy Kit (Qiagen, Venlo, Netherlands). Then, a MiniAmp Plus Thermal Cycler (Applied Biosystems, Waltham, MA, USA) was used for reverse transcription and detection of miRNAs. From miRNAs, cDNA was synthesized using an HB miR Multi Assay Kit (HEIMBIOTEK, Seongnam, Korea), and qPCR was then performed by using a QuantStudio 5 (Applied Biosystems). Relative miRNA expression was calculated using the comparative 2^−ΔΔCt^ method and normalized to miRNA 39-3p expression.

### Human peripheral ILC isolation and stimulation

A human pan-ILC enrichment kit (STEMCELL Technologies, Vancouver, Canada) was used to purify ILCs from peripheral blood mononuclear cells. Isolated ILCs were identified by flow cytometric analysis using the following antibodies: anti-lineage (BD Biosciences), anti-CD45 (BD Biosciences), and anti-CD127 (BioLegend). The expression levels of GATA-3 (Abcam), ROR-γt (Invitrogen, Waltham, MA, USA), and IL-22 (Abcam) in ILCs were investigated by western blot analysis. Then, 1 × 10^3^ ILCs were cocultured with 1 × 10^5^ monocytes (with or without hsa-miR-4517 transfection) seeded in a 48-well plate in RPMI 1640 medium supplemented with 2% fetal bovine serum. These cells were stimulated with 1 μg of LPS for 24 h.

### Human peripheral neutrophil activation and migration

For neutrophil isolation, the layer containing granulocytes was separated and placed in Hanks’s balanced salt solution with 2 mmol/L ethylenediaminetetraacetic acid and 2% dextran for 20 min. After the removal of red blood cells by hypotonic lysis, neutrophils were isolated using a neutrophil isolation kit (Miltenyi Biotec). For neutrophil stimulation, the cells were seeded (1 × 10^6^) and treated with 1 µM Dex or 1 μg/mL MlEVs in the presence of 10 μg/mL LPS for 24 h. For transfection, 50 nM synthetic miRNA mimics were transfected into neutrophils treated with 10 ng/mL IL-1β (R&D Systems). The levels of MPO in the supernatants were evaluated using an ELISA kit (R&D Systems). For the migration assay, the cells were stained with 2 μmol of calcium aceto-methyl ester (Life Technologies, Eugene, OR, USA) for 30 min at 37 °C and washed with 1× HBSS. Then, the cells (1 × 10^5^) were seeded in the upper chamber with a pore size of 3.0 μm (Neuro Probe, Gaithersburg, MD, USA). Phenol red-free RPMI medium containing ILCs (1 × 10^3^) was added to the lower chamber, and the Transwell plate was incubated for 2 hours at 37 °C. The signal was measured at excitation and emission wavelengths of 480 and 520 nm, respectively, with a fluorescence microplate reader (BioTek).

### Statistical analysis

All statistical analyses were performed using IBM SPSS software, version 26.0 (IBM Corp., Armonk, NY, USA). GraphPad Prism 8.0 software (GraphPad Inc., San Diego, CA, USA) was used to create graphs.

## Results

### Lower levels of MlEV-specific IgG4 but not IgG1 in asthmatic patients

**Fig. 1:**
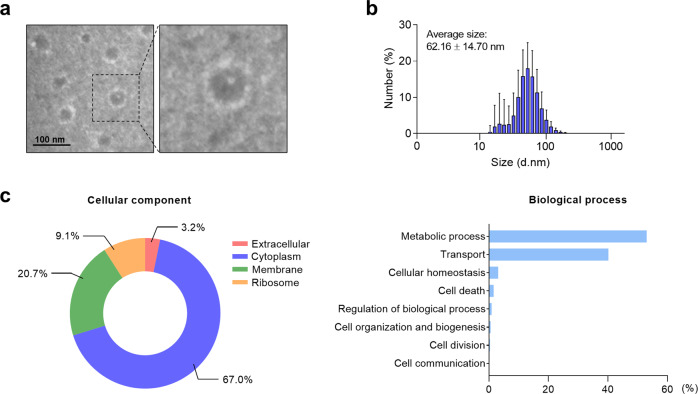
Characterization and identification of *Micrococcus luteus*-derived extracellular vesicles. **a** Images of MlEVs observed using transmission electron microscopy. **b** EV size measured using a dynamic light-scattering analyzer. **c** Protein profiles in EVs identified according to cellular components or biological processes. MlEV *Micrococcus luteus*-derived extracellular vesicle.

**Fig. 2:**
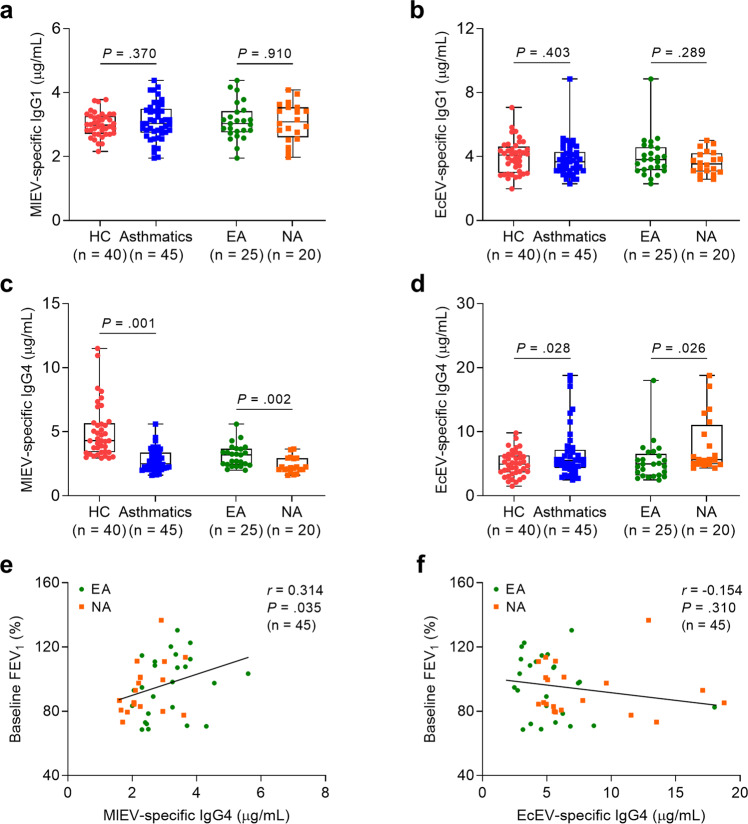
The prevalence of serum EV-specific IgG1 and IgG4 in asthmatic patients. Comparisons of the levels of **a** MlEV- and **b** EcEV-specific IgG1 as well as **c** MlEV- and **d** EcEV-specific IgG4 antibodies in serum between asthmatic patients and HCs and between patients with eosinophilic asthma and those with neutrophilic asthma. Data are presented in box plots. *P* values were obtained by Student’s *t*-test. Correlations of **e** MlEV- or **f** EcEV-specific IgG4 with FEV_1_ (%) values in asthmatic patients. Data are presented as the Pearson correlation coefficient *r* (*P* value). EA eosinophilic asthma, EcEV *Escherichia coli*-derived extracellular vesicle, FEV_1_ forced expiratory volume in 1 s, HCs healthy control subjects, NA neutrophilic asthma.

To evaluate the abundance of MlEVs in the serum of the study subjects, *M. luteus* was cultured, and its EVs were isolated. When MlEVs were investigated using transmission electron microscopy, they showed a spherical lipid bilayered morphology with an average diameter of 62.16 ± 14.70 nm (Fig. 1a, b). Moreover, these EVs contained various proteins related to cellular components and biological processes (Fig. 1c). The most abundant proteins in the EVs are shown in Supplementary Table 1. Then, we recruited the study subjects, and their clinical characteristics are depicted in Supplementary Table 2. Compared to HCs, asthmatic patients had lower baseline FEV_1_ (%) but higher total eosinophil counts and IgE, ECP, and MPO levels (*P* < 0.05 for all). Among the patients, baseline FEV_1_ (%) was not significantly different between NA and EA; however, levels of MPO were higher in NA, while total eosinophil count and IgE and ECP levels were higher in EA. Although the levels of MlEV- or EcEV-specific IgG1 in serum were not significant between the groups (Fig. 2a, b), patients with NA showed the lowest levels of MlEV-specific IgG4 but the highest levels of EcEV-specific IgG4 (Fig. 2c, d). Moreover, levels of IgG4 specific to MlEVs but not to EcEVs were positively correlated with baseline FEV_1_ (%) (Fig. 2e, f), indicating that the decreased levels of MlEV-specific IgG4 (the prevalence of MlEVs) may be associated with lower lung function in asthmatic patients.

### Inhibition of neutrophilic inflammation by MlEV treatment in mice

**Fig. 3:**
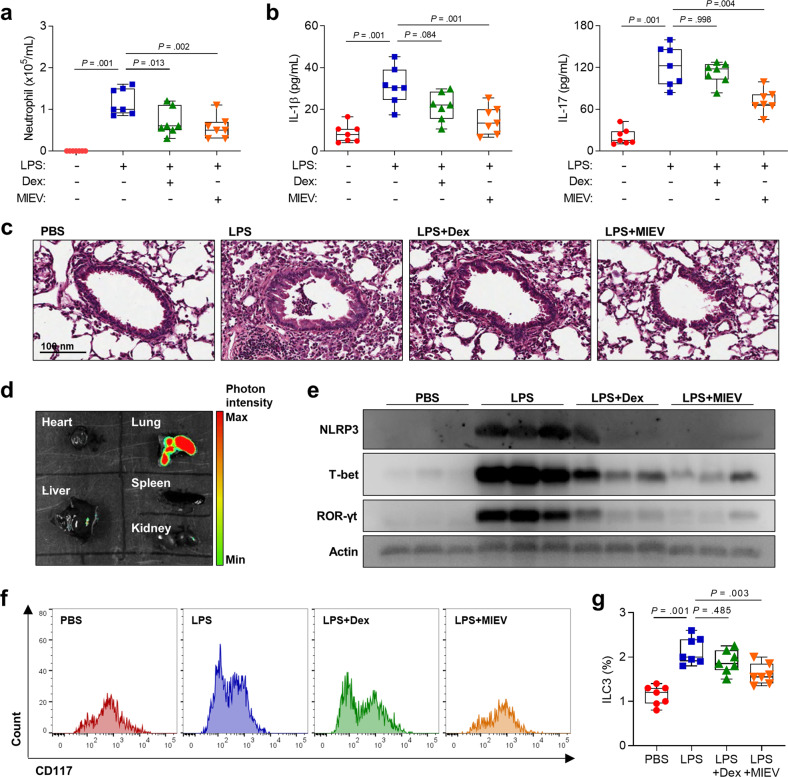
Therapeutic potential of MlEVs in a mouse model of neutrophilic asthma. Neutrophil counts **a** and of IL-1β and **b** IL-17 levels in the bronchoalveolar lavage fluid. Data are presented in box plots, *n* = 7. *P* values were obtained by one-way ANOVA with Bonferroni’s post hoc test. **c** Histological analysis of lung tissues stained with hematoxylin and eosin. **d** Fluorescence assay for detecting MlEVs in dissected organs. **e** Expression of NLRP3, T-bet, and ROR-γt in lung tissues. **f** Flow cytometric histograms of CD117-positive ILCs. **g** Frequencies of ILC3s in lung tissues. Data are presented in box plots, *n* = 7. *P* values were obtained by one-way ANOVA with Bonferroni’s post hoc test. Dex dexamethasone, ILC3s group 3 innate lymphoid cells, LPS lipopolysaccharide, MlEV *Micrococcus luteus*-derived extracellular vesicle, NLRP3 NOD-, LRR- and pyrin domain-containing protein 3, PBS phosphate-buffered saline, ROR-γt retinoic acid receptor-related orphan nuclear receptor gamma t, T-bet T-box expressed in T cells.

When LPS-induced asthmatic mice were treated with MlEVs, the EVs significantly decreased the number of neutrophils (but not eosinophils) and the production of IL-1β/IL-17 in the BALF. Although Dex also attenuated inflammation, MlEVs could more effectively reduce neutrophilic airway inflammation (Fig. 3a, b and Supplementary Fig. 1). In the lung tissues, reduced immune cell infiltration and epithelial thickness were also noted (Fig. 3c). By using Cy7 mono NHS ester-labeled MlEVs, the present study clarified that these EVs could affect the lungs without influencing other organs when mice were intranasally treated (Fig. 3d). Moreover, the expression of NLRP3, T-bet, and ROR-γt was significantly suppressed by MlEV treatment in the lung tissues of asthmatic mice (Fig. 3e). As such transcription factors are related to T cells and ILCs, we further investigated lung ILC3s by flow cytometric analysis in asthmatic mice and found that, compared to Dex, MIEVs could reduce the proportion of ILC3s (Supplementary Fig. 2 and Fig. 3f, g).

### Effects of MlEVs on pathophysiological conditions in AECs

**Fig. 4:**
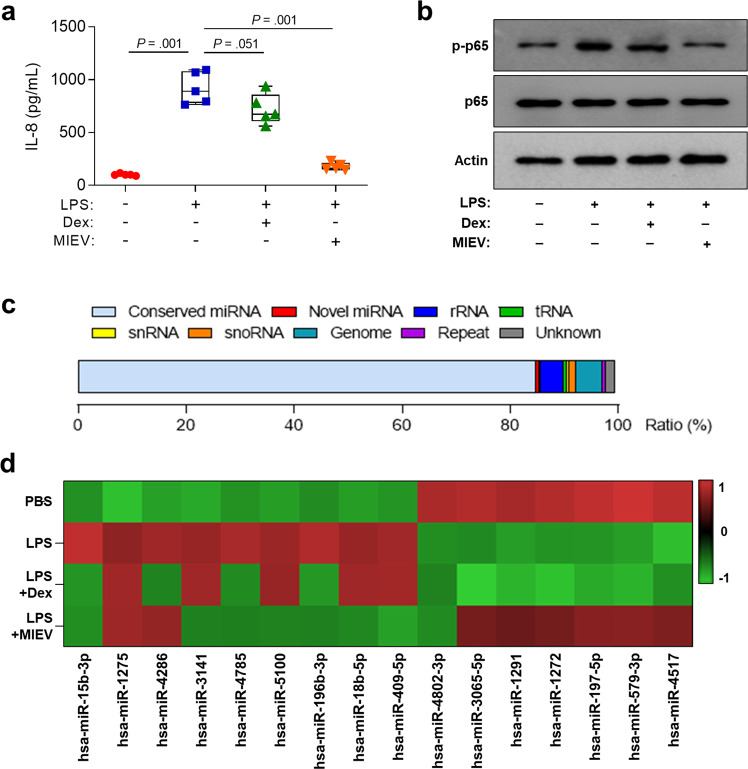
The effects of MlEVs on airway epithelial cells. **a** Levels of IL-8 released from A549 cells followed by treatment with Dex or MlEVs in the presence of LPS. Data are presented in box plots, *n* = 5. *P* values were obtained by one-way ANOVA with Bonferroni’s post hoc test. **b** Phosphorylation of p65 in the cells. **c** RNA composition in the cells. **d** Expression of miRNAs in the cells evaluated by microarray analysis.

To investigate the mechanism by which MlEVs regulate airway inflammation, AECs were treated with or without MlEVs in the presence of LPS. These EVs markedly reduced the production of IL-8 as well as the phosphorylation of p65 in A549 cells (Fig. 4a, b). Moreover, the current study showed that miRNAs were highly expressed in the cells, and their expression was significantly changed by MlEVs (Fig. 4c, d). In particular, in the presence of LPS, MlEVs partially restored miRNA expression to the physiological state via downregulation of 7 miRNAs (hsa-miR-15b-3p, hsa-miR-3141, hsa-miR-4785, hsa-miR-5100, hsa-miR-196b-3p, hsa-miR18b-5p, and hsa-miR-409-5p) and upregulation of 6 miRNAs (hsa-miR-3065-5p, hsa-miR-1291, hsa-miR-1272, hsa-miR-197-5p, hsa-miR579-3p, and hsa-miR-4517) in the cells. Here, we selected these 6 upregulated miRNAs as target genes for suppressing airway inflammation.

### Effects of miRNAs on IL-1β-producing monocytes

**Fig. 5:**
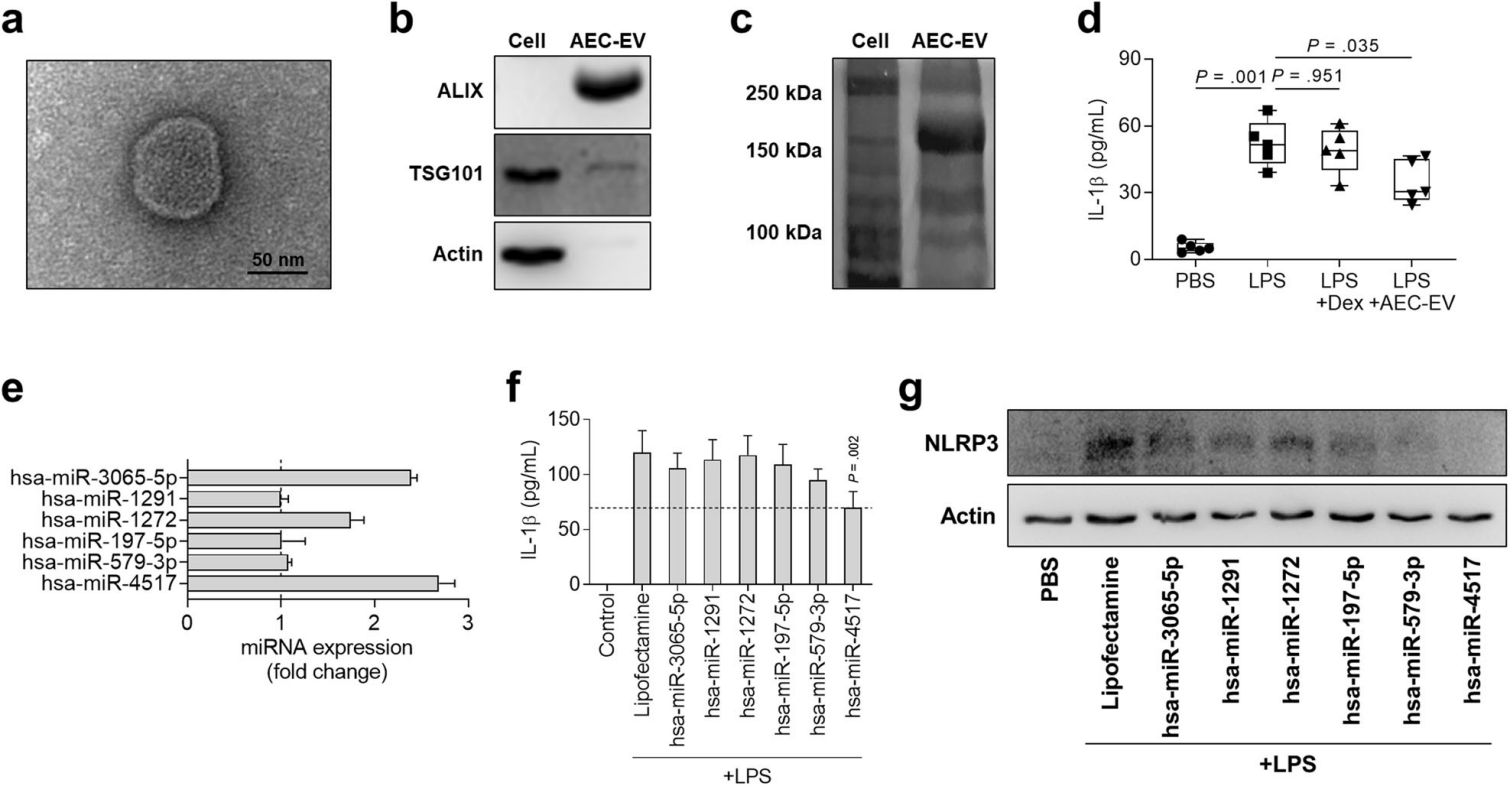
The effects of AEC-EVs containing miRNAs in monocytes. **a** Image of AEC-EVs. **b** Expression of EV markers. **c** Protein patterns in EVs. **d** Effect of AEC-EVs on monocyte activation in the presence of LPS. **e** Relative expression of miRNAs in EVs. **f** Levels of IL-1β released from monocytes of asthmatic patients. Data are represented as the means ± SDs, *n* = 5. The *P* value was obtained by Student’s *t*-test, compared to the Lipofectamine-treated group. **g** Expression of NLRP3 in monocytes. AEC-EV airway epithelial cell-derived extracellular vesicle, ALIX ALG-2-interacting protein X, PBS phosphate-buffered saline, TSG101 tumor susceptibility 101.

Because miRNAs are known to be contained in mammalian EVs, AEC-EVs were further isolated and identified. Similar to other EVs, AEC-EVs were lipid bilayered vesicles with a spherical shape (Fig. 5a) and strongly expressed ALIX, whereas A549 cells expressed TSG101 and actin (Fig. 5b). Moreover, the protein band patterns were different between AECs and AEC-EVs (Fig. 5c). Then, we investigated the effect of AEC-EVs on monocyte activation since these immune cells are important for neutrophilic inflammation. AEC-EVs significantly inhibited IL-1β production by monocytes of asthmatic patients (Fig. 5d). To investigate the importance of miRNAs in AEC-EVs, we analyzed the expression of 6 target miRNAs and found that these miRNAs were highly expressed in the EVs (Fig. 5e). Among them, hsa-miR-4517 markedly reduced IL-1β production and NLRP3 expression in monocytes stimulated with LPS (Fig. 5f, g). However, MlEVs or these target miRNAs could not significantly suppress neutrophil activation and MPO release (Supplementary Fig. 3a, b), suggesting that AEC-EVs could contribute to the inhibition of neutrophil airway inflammation by regulating monocyte activation.

### Decreased hsa-miR-4517 expression and increased ILC3 activation

**Fig. 6:**
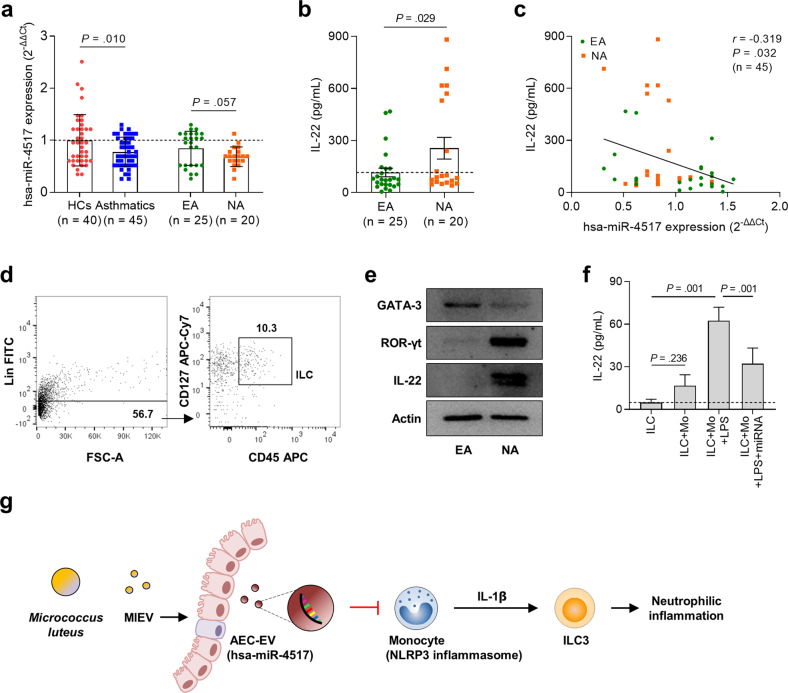
Association between miRNAs in plasma and inflammatory status in asthmatic patients. **a** Expression of miRNAs in the plasma of asthmatic patients compared to HCs. **b** Levels of IL-22 in plasma in comparison between patients with EA and those with NA. Data are represented as the means ± SDs. *P* values were obtained by Student’s *t*-test. **c** Correlation between miRNA expression and IL-22 levels. Data are presented as the Pearson correlation coefficient *r* (*P* value). **d** Gating strategy of ILC isolation. **e** Expression of GATA-3, ROR-γt, and IL-22 in ILCs. **f** IL-22 levels released from ILCs treated with or without MlEVs in the presence of monocytes. Data are represented as the means ± SDs. *P* values were obtained by Student’s *t* test. **g** Schematic of MlEVs inhibiting airway inflammation. ILC innate lymphoid cell, GATA3 GATA-binding protein 3, Mo monocyte.

In the present study, miRNA expression was investigated in human plasma according to the inflammatory phenotype of asthma. Lower hsa-miR-4517 expression was noted in asthmatic patients than in HCs. Although statistical significance was not noted, the expression of hsa-miR-4517 tended to be more markedly decreased in patients with NA than in those with EA (Fig. 6a). Moreover, the levels of IL-22 in plasma were significantly higher in patients with NA than in those with EA (Fig. 6b). In particular, the expression of hsa-miR-4517 had a negative correlation with the levels of IL-22 in plasma (Fig. 6c). To confirm the association between miRNA expression and IL-22 production, ILCs were isolated from human peripheral blood mononuclear cells (Fig. 6d). ILCs from patients with NA showed significantly higher expression of ROR-γt and IL-22 but lower expression of GATA-3 than those from patients with EA (Fig. 6e). In the presence of monocytes stimulated with LPS, ILCs robustly released IL-22, but this IL-22 release was significantly decreased when monocytes transfected with hsa-miR-4517 were cocultured (Fig. 6f). Moreover, elevated ILC counts were associated with increased neutrophil transmigration (Supplementary Fig. 4). Here, we propose a possible mechanism of action in which MlEVs contribute to attenuating NA (Fig. 6g).

## Discussion

This is the first study to demonstrate the clinical significance of decreased levels of MlEV-specific IgG4 in asthmatic patients in association with lower lung function. Moreover, the effect of MlEVs on airway inflammation by regulating miRNAs in AECs was shown to suppress monocyte function, resulting in ILC3 inactivation. In particular, the present study showed that hsa-miR-4517 plays a pivotal role in innate immune responses to attenuate neutrophilic inflammation. These findings suggest EVs as a key mediator linking bacterial activity to the host immune system, where MlEVs influence the modulation of immune responses in NA.

To date, metagenomic data analyses have identified distinct microbial compositions according to asthma phenotypes^33^. For example, asthmatic patients with a neutrophilic phenotype showed low diversity of their airway microbiome and a pathogenic bacterial burden^9^. In particular, EVs are novel molecules released from bacteria and found in human biofluids^21^. Previously, asthmatic patients have been shown to be more significantly sensitized to bacterial EVs from *Enterobacter cloacae*, *Pseudomonas aeruginosa*, and *Staphylococcus aureus* with higher levels of EV-specific IgG in their serum^20^. However, levels of IgG4 specific to *Lactococcus lactis*-derived EVs were significantly lower in patients with allergic asthma than in HCs^34^. Since IgG4 antibody is known as a soluble marker of repeated and long-term exposure to external molecules in a noninfectious setting^35^, EV-specific IgG4 levels could be analyzed as a serum biomarker for diagnosing asthma rather than for performing metagenomic analysis, which is time- and cost-consuming. Our recent study revealed a lower abundance of MlEVs in serum from asthmatic patients than in serum from HCs^22^. Based on this finding, we further verified that the levels of serum MlEV-specific IgG4 were significantly lower in patients with NA than in those with EA and were positively correlated with FEV_1_ (%) values. These findings may indicate that patients with NA are less frequently exposed to MlEVs than those with EA. Therefore, exposure to MlEVs may be one of the environmental factors determining the neutrophilic phenotype in asthmatic individuals.

Although innate immunity has been less commonly discussed in asthma pathogenesis, recent studies have suggested its association with antigen-independent inflammatory responses^36^. In particular, AECs are critical in host defense for initiating the innate immune response with the activation of NF-κB pathways via toll-like receptors, leading to the production of proinflammatory cytokines^37–39^. The present study demonstrated that MlEVs could inhibit the phosphorylation of p65, which contributes to the secretion of chemoattractants for neutrophils. Moreover, circulating miRNAs within a type of EVs were noted in human blood. In particular, hsa-miR-4517 suppressed NLPR3 inflammasome activation and IL-1β production in monocytes. However, the expression of hsa-miR-4517 was downregulated in plasma from patients with NA. Therefore, hsa-miR-4517 may play a key role in mediating innate immune crosstalk between AECs and monocytes via an efficient EV delivery system.

A previous study showed that miRNAs could be potential therapeutic agents to modulate airway inflammation related to specific receptors or cytokines in asthma^40^. For years, many studies have focused on the dysregulation of miRNA expression in AECs and immune cells of asthmatic patients^41^. Therefore, miRNAs could be a stable marker for classifying asthma phenotypes and understanding their molecular mechanisms. In particular, such miRNA levels were identified to correlate with eosinophil counts and relevant clinical parameters, including ECP levels and FVC^42^. Moreover, several miRNAs have been found to circulate within biofluids within EVs^43^. The present study demonstrated that hsa-miR-4517 within AEC-EVs circulated in the blood, finally contributing to innate immunity through its protective function. Thus, hsa-miR-4517 could be a potential biomarker and a therapeutic target for NA. Nevertheless, further validation studies are needed in larger cohorts, as effective miRNAs in mice have not yet been clarified since miRNAs in humans were not matched to those in mice.

In patients with NA, the intervention of Th17 cells with elevated levels of IL-17 in sputum and serum has been reported^44,45^. IL-17 is known to recruit and activate neutrophils in the airways, leading to uncontrolled symptoms and steroid resistance in asthma^46^. In particular, ILC3s have been identified as one of the cellular sources of both IL-17 and IL-22. ILC3s are divided into 2 subtypes depending on their expression of the NCR NKp46, and NCR^-^ ILC3s are known to predominantly produce IL-17 in the presence of IL-1β^47^. Although these cells are less clearly defined in asthma, recent studies have revealed the contribution of ILC3s to lung dysfunction and asthma severity^48^. Moreover, the proliferation of IL-17-producing ILC3s induced by NLRP3 activation in the lungs was suggested to directly drive airway hyperresponsiveness, which was ameliorated by the blockade of IL-1β^49^. Although MlEVs could not directly inhibit neutrophil activation, the present study showed that MlEVs significantly reduced the levels of IL-17 in the BALF and the proportion of ILC3s as well as the expression of their transcription factors (ROR-γt) in the lungs of asthmatic mice, which were the result of NLRP3 inactivation and IL-1β reduction in monocytes.

In conclusion, these findings suggest that MlEVs can be a new therapeutic target for neutrophilic airway inflammation by regulating miRNA expression in AECs, leading to the suppression of innate immune responses in NA.

## Supplementary information

Supplementary Information

Supplementary Information (not for publication)

## Data Availability

The data that support the findings of this study are available from the corresponding author upon reasonable request.
