# Supplementary material for: Micrococcus luteus-derived extracellular vesicles attenuate neutrophilic asthma by regulating miRNAs in airway epithelial cells
Source: Exp Mol Med. 2023 Jan 13;55(1):196–204. doi: 10.1038/s12276-022-00910-0 (PMC9898544; doi:10.1038/s12276-022-00910-0)
Supplement: Supplementary file 1 — Supplementary Information [file 12276_2022_910_MOESM1_ESM.pdf]

Supplementary Information

***Micrococcus luteus*-derived extracellular vesicles attenuate neutrophilic asthma by regulating miRNAs in airway epithelial cells**

Soyoon Sim,[1,2] Dong-Hyun Lee,[1,2] Kwang-sun Kim,[3] Hyeon Ju Park,[4] Yoon-Keun Kim,[4] Youngwoo Choi,[1*] Hae-Sim Park[1,2*]

[1]Department of Allergy and Clinical Immunology, Ajou University School of Medicine, Suwon, Korea

[2]Department of Biomedical Sciences, Graduate School of Ajou University, Suwon, Korea

[3]Department of Chemistry and Chemistry Institute for Functional Materials, Pusan National University, Busan, Korea

[4]MD Healthcare Inc., Seoul, Korea

*These authors equally contributed to this work

Corresponding authors:

Youngwoo Choi PhD

Department of Allergy and Clinical Immunology, Ajou University School of Medicine, 164 Worldcup-ro, Suwon, Korea

Tel: +82-31-219-4277,

Fax: +82-31-219-4407,

Email: cyw3789@gmail.com

Hae-Sim Park MD, PhD

Department of Allergy and Clinical Immunology, Ajou University School of Medicine, 164

Worldcup-ro, Suwon, Korea

Tel: +82-31-219-5196,

Fax: +82-31-219-5154,

Email: hspark@ajou.ac.kr

Supplementary Table 1: Proteomic analysis of 10 most abundant MlEV components

Description	Ensembl Gene ID	Q-value: Combined	Sum PEP score	MW [kDa]	Coverage [%]	# Peptides	# PSM	# Unique Peptides
Extracellular solute-binding protein	Mlut_15570	0	679.165	66.6	75	52	1455	52
Formaldehyde dehydrogenase	Mlut_04320	0	388.074	42.6	73	24	741	24
Subtilase family protease	Mlut_02600	0	435.808	101.2	51	41	666	41
ABC-type Fe3+-hydroxamate transport system, periplasmic component	Mlut_22080	0	299.04	37.5	84	19	558	19
Isocitrate dehydrogenase [NADP]	Mlut_04530	0	978.427	79.8	81	68	745	68
Dihydrolipoyl dehydrogenase	Mlut_13320	0	707.000	48.9	75	47	652	47
5'-nucleotidase/2',3'-cyclic phosphodiesterase-like hydrolase	Mlut_03620	0	486.902	77.4	52	29	316	29
Acetyl-coenzyme A synthetase	Mlut_18230	0	469.481	71.0	47	32	463	32
ABC-type dipeptide transport system, periplasmic component	Mlut_01730	0	267.917	60.6	53	20	184	20
Glyceraldehyde-3-phosphate dehydrogenase	Mlut_11350	0	350.721	36.7	80	24	412	24

ABC, ATP-binding cassette; MlEV, *Micrococcus luteus*-derived extracellular vesicles; MW, molecular weight; PEP, posterior error probability; PSM, peptide-spectrum match.

Supplementary Table 2: Demographic data of the study subjects according to asthma phenotypes

Variables	HCs (n = 40)	Asthmatics (n = 45)	*P* value	EA (n = 25)	NA (n = 20)	*P* value
Age (y)	41.1 ± 16.1	44.0 ± 17.6	.782	38.2 ± 16.6	44.8 ±15.0	.180
Female sex (%)	47.5	55.6	.458	56.0	55.0	.947
Atopy (%)	47.5	55.6	.173	72.0	50.0	.130
Severe asthma (%)	ND	62.2	ND	16.0	10.0	.556
Baseline FEV1 (%)	106.3 ± 12.9	13.3	.004	95.8 ± 19.1	93.2 ± 15.7	.632
PC20 (mg/mL)	ND	94.7 ± 17.5	ND	5.2 ± 6.3	5.7 ± 2.9	.759
Sputum Eos (%)	ND	11.6 ± 16.5	ND	19.4 ± 18.1	0.8 ± 0.9	.001
Sputum Neu (%)	ND	54.5 ± 30.1	ND	34.3 ± 24.7	79.8 ± 11.0	.001
Total IgE (IU/mL)	102.4 ± 93.1	511.6 ± 527.2	.001	544.6 ± 544.8	248.3 ± 269.1	.025
ECP (ng/mL)	38.6 ± 52.8	412.9 ± 470.5	.003	211.9 ± 230.8	42.6 ± 66.7	.002
MPO (ng/mL)	198.2 ± 89.1	273.8 ± 109.8	.019	223.6 ± 83.9	339.8 ± 184.6	.045

Values are given as n (%) for categorical variables and as mean ± SD for continuous variables.

P values were applied by Pearson chi-square test for categorical variables and Student's *t* test for continuous variables.

HCs, healthy control subjects; EA, eosinophilic asthma; NA, neutrophilic asthma; FEV1, forced expiratory volume in 1 s; methacholine PC20, the provocative concentration of methacholine required to cause a 20% fall in FEV1; Eos, eosinophils; Neu, neutrophils; IgE, immunoglobulin E; ECP, eosinophil cationic protein; MPO, myeloperoxidase; ND, not data.

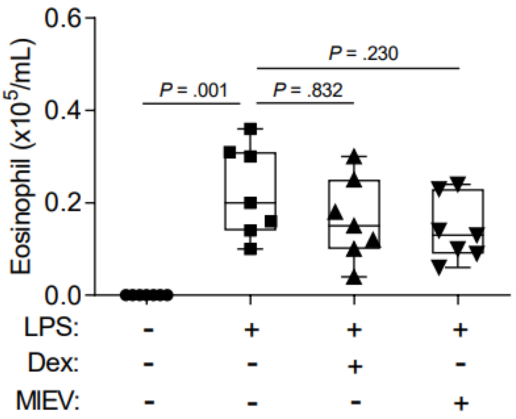

Supplementary Fig. 1 Eosinophil numbers in the bronchoalveolar lavage fluid of mice. Data are presented as box plots, n = 7. *P* values were obtained by one-way ANOVA with Bonferroni's *post hoc* test. Dex, dexamethasone; LPS, lipopolysaccharide; MlEV, *Micrococcus luteus*-derived extracellular vesicle.

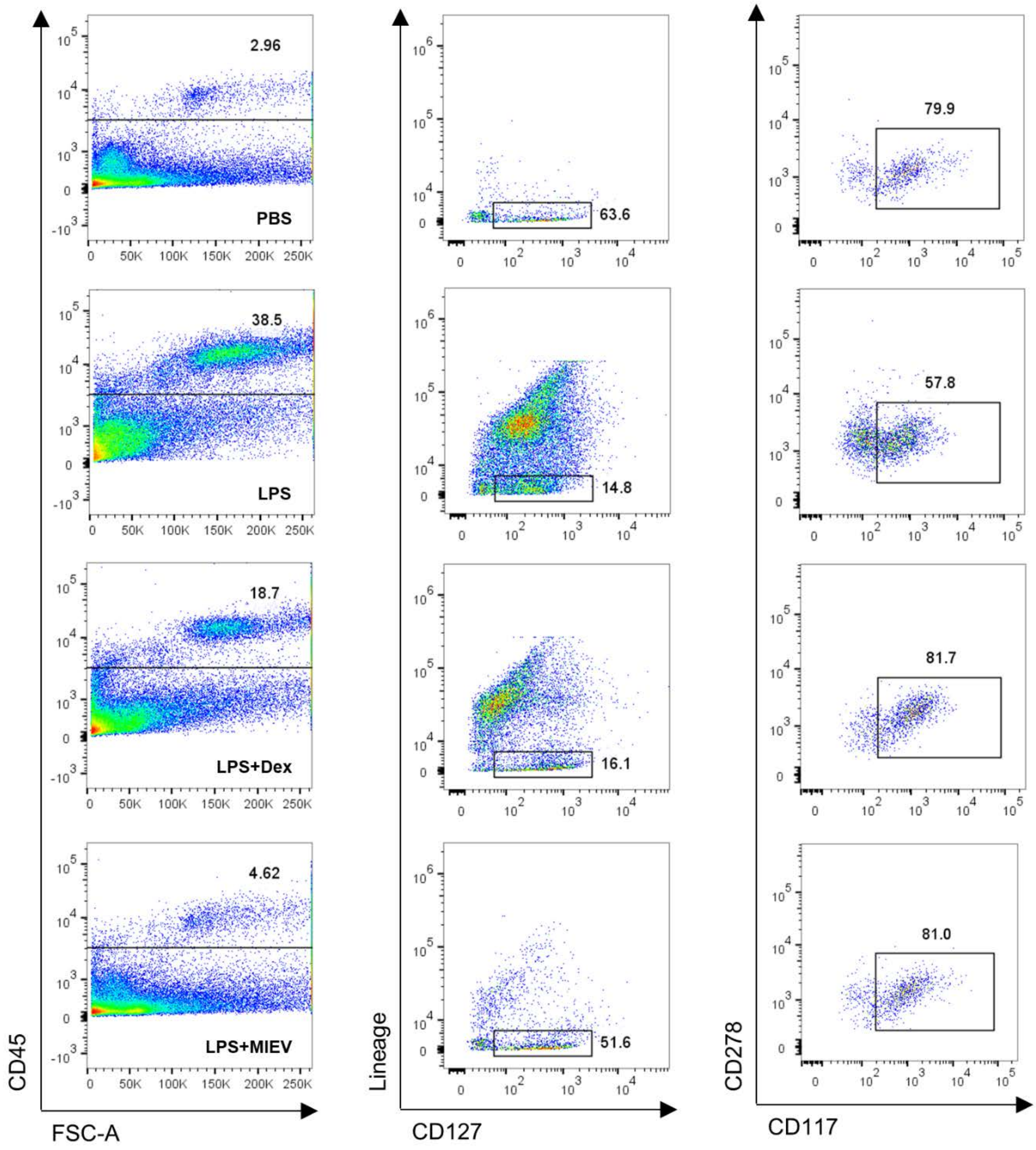

Supplementary Fig. 2 Quantification of ILC3s in the lung tissues of mice. The cells were defined by expression of CD45, CD127/IL-7Rα, and CD117/c-kit, but lack of lineage markers and CD278/ICOS. PBS, phosphate buffered saline.

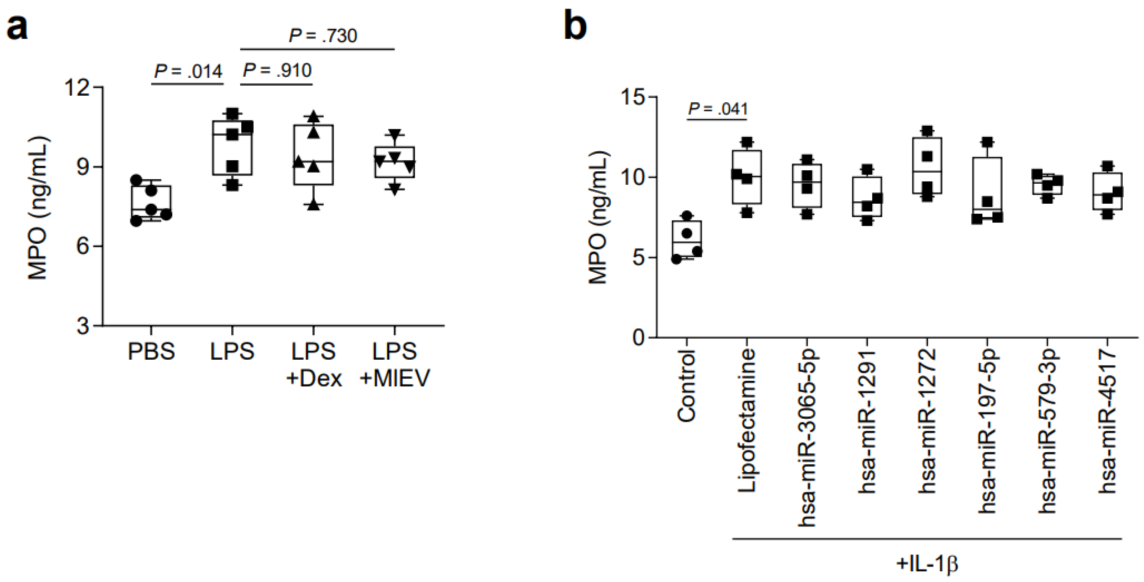

Supplementary Fig. 3 Effect of MlEVs or MiRNAs on human peripheral neutrophils. a Levels of MPO in culture supernatants of neutrophils treated with Dex or MlEV in the presence of LPS. Data are presented as box plots, n = 5. *P* values were obtained by one-way ANOVA with Bonferroni's *post hoc* test. **b** Levels of MPO in neutrophils transfected with various miRNAs in the presence of IL-1β. Data are presented as box plots, n = 4. *P* values were obtained by one-way ANOVA with Bonferroni's *post hoc* test. MPO, myeloperoxidase.

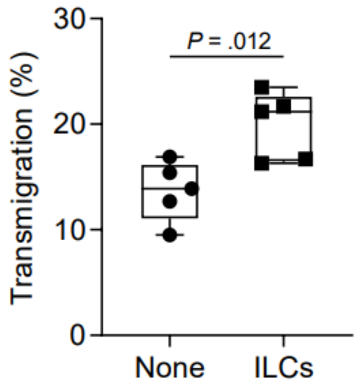

Supplementary Fig. 4 The percentage of migrating neutrophils in the presence of ILCs.

Data are presented as box plots, n =5. *P* values were obtained by Student's *t* test.
