# Supplementary material for: Micrococcus luteus-derived extracellular vesicles attenuate neutrophilic asthma by regulating miRNAs in airway epithelial cells
Source: Exp Mol Med. 2023 Jan 13;55(1):196–204. doi: 10.1038/s12276-022-00910-0 (PMC9898544; doi:10.1038/s12276-022-00910-0)

**Supplementary Information**

**(not for publication)**

***Micrococcus luteus*-derived extracellular vesicles attenuate neutrophilic asthma by regulating miRNAs in airway epithelial cell**

Soyoon Sim,^1,2^ Dong-Hyun Lee,^1,2^ Kwang-sun Kim,^3^ Hyeon Ju Park,^4^ Yoon-Keun Kim,^4^ Youngwoo Choi,^1*^ Hae-Sim Park^1,2*^

^1^Department of Allergy and Clinical Immunology, Ajou University School of Medicine, Suwon, Korea

^2^Department of Biomedical Sciences, Graduate School of Ajou University, Suwon, Korea

^3^Department of Chemistry and Chemistry Institute for Functional Materials, Pusan National University, Busan, Korea

^4^MD Healthcare Inc., Seoul, Korea

*These authors equally contributed to this work

**Corresponding authors:**

Youngwoo Choi PhD

Department of Allergy and Clinical Immunology, Ajou University School of Medicine, 164 Worldcup-ro, Suwon, Korea

Tel: +82-31-219-4277,

Fax: +82-31-219-4407,

Email: cyw3789@gmail.com

Hae-Sim Park MD, PhD

Department of Allergy and Clinical Immunology, Ajou University School of Medicine, 164 Worldcup-ro, Suwon, Korea

Tel: +82-31-219-5196,

Fax: +82-31-219-5154,

Email: hspark@ajou.ac.kr

**Fig. 2b**


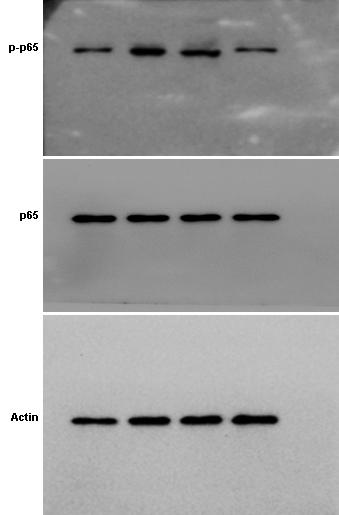


**Fig. 4b**


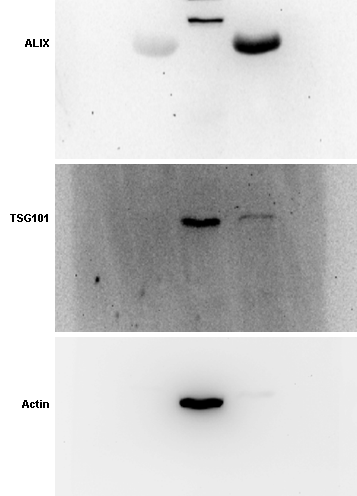


**Fig. 4g**


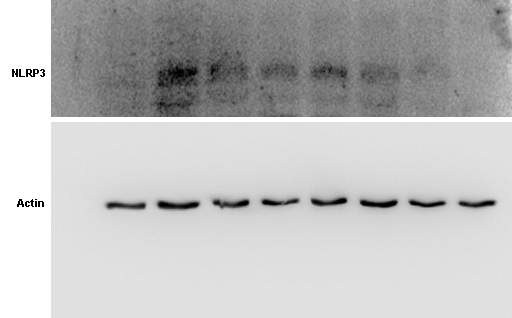


**Fig. 5b**


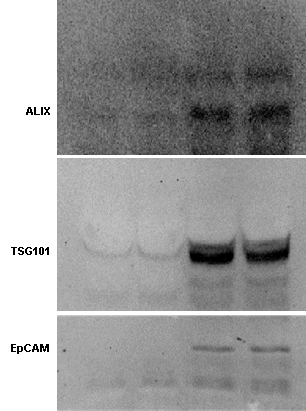


**Fig. 5h**


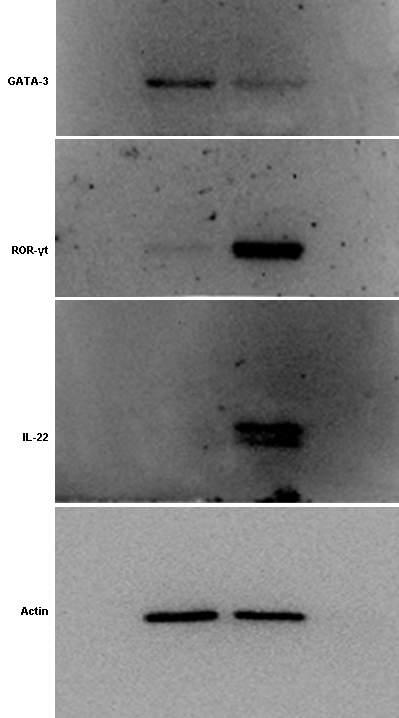


**Fig. 6e**


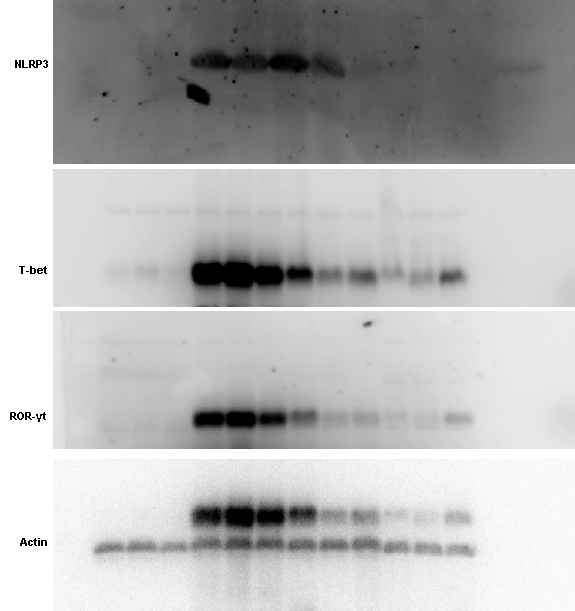

Supplement: Supplementary file 2 — Supplementary Information (not for publication) [file 12276_2022_910_MOESM2_ESM.docx]
